# COVID-19 in Ethiopia: current situation, missed opportunities, and the risk of health system disruptions

**DOI:** 10.11604/pamj.supp.2020.35.2.23906

**Published:** 2020-06-08

**Authors:** Sibhatu Biadgilign, Muluneh Yigzaw

**Affiliations:** 1 Public Health Research Consultant, Addis Ababa, Ethiopia

**Keywords:** COVID-19, health system, response, cases, Ethiopia

## Abstract

COVID-19 started in Wuhan province of china and spread in a faster rate covering all continents in the world. The pandemic has affected the socio-economic, political fabric of all countries, and exacerbated undernutrition and food insecurity problems of Low and Middle Income Countries (LMICs). COVID-19 has also disrupted the health system, resulted in low level utilization of essential services, such as childhood immunization, labor and childbirth, and treatment for children with serious illness. Unless the spread of the COVID-19 pandemic slows down, Ethiopia and other low income countries are at the verge of losing all the progresses made in health and wellbeing over the last two decades.

## Commentary

The novel corona virus disease 2019 (COVID-19) which originated in Wuhan, China, in December 2019, spread rapidly around the world and became a pandemic [1]. COVID-19 was reported in Africa for the first time in Egypt on February 14, 2020 [2] and as well the initial cases were imported from other regions [3]. In Ethiopia, the first COVID-19 case was reported on 13 March 2020. As of June 08, 2020, Ethiopia reported a total of 2156 COVID-19 cases and 27 deaths. After the first COVID-19 case was reported, the government´s response was swift, but short-lived. A range of strategies was devised and implemented to curb further spread of the pandemic: including quarantine of people coming from abroad at least for 14 days, closure of schools, suspending of public gatherings, and temporary closure of churches and mosques [4]. A state of emergency was also declared and people were advised to avoid non-essential travel. During the first 53 days after the index case was reported, the number of COVID-19 cases were modest with an average number of three cases per day, an indication that the government´s initial mitigation strategy was working. After 53 days, however, the case load sharply increased. On the 54th day, the Ministry of Health reported 17 COVID-19 cases just in one day followed by 29 cases on the 55th day, 19 of whom with no contact history to a known COVID-19 case. This huge number of cases with no contact history to a known case is worrying as this is a clear evidence of the resurface of SARS-Cov-2 community level transmission in Ethiopia.

The sudden increase of COVID-19 cases after 53 days of reporting of the index case was perhaps the result of the government´s inability to sustain its initial mitigation strategies. Citizens coming from high caseload neighboring countries have entered to Ethiopia without proper vetting as the government has loosened its restrictions on in-land transportations. As time went, local governments failed to apply other mitigation strategies as well. In countries such as Ethiopia, stay at home prevention measure is difficult to apply for a prolonged time as significant numbers of people are deployed in the informal sector. It is barely possible to force these citizens to stay at home without providing food and other essential items. Furthermore, low level awareness among the public about the risk of acquiring the virus and its mode of transmission was an exacerbating factor. The Ethiopian healthcare system lacks adequate resources to manage severe cases unless the spread of the virus is slowed down [5]. For an estimated population of over 108 million, Ethiopia has 450 respirators. Out of it, only 54 have reserved for COVID-19 patients. As the health system wreaks havoc by multiple COVID-19 cases, other essential health services will also be affected. The surveillance for other epidemic prone disease will also be weakened and death surge from outbreaks of other contagious diseases is imminent. The health care for maternal, newborn and child health will be compromised, and women will be forced to give birth at home without a skilled birth attendant. Children will not able to receive vaccines on the scheduled dates and vaccine preventable disease will be resurfaced. Lack of adequate care for children coupled with undernutrition and food insecurity [6] reduced food production leading to food shortages in the country. In addition to this, on recent incidence the damage of crop production from desert locust will be another fueling factor for complications and death among children less than five years of age.

In order to curb the spread of the pandemic, Ethiopia should ramp up its mitigation strategies at borders with a huge influx of refugees. The country should establish examination and testing centers at borders with Kenya, Djibouti and Somalia as the number of reported COVID-19 cases are steadily increasing in these countries. The Addis Ababa-Djibouti transport corridor is also warranted a strong mitigation strategy as there are a huge influx of traders and long truck drivers. Unless the spread of the COVID-19 pandemic slows down, Ethiopia and other low income countries are at the verge of losing all the progresses in health and wellbeing. This is the great setback of the health system in low income countries with significant reversal of gains made over the last two decades.

## Competing interests

The authors declare no competing interests.
